# Irrigation during concomitant bipolar ablation: does it matter?

**DOI:** 10.1186/1749-8090-8-S1-O55

**Published:** 2013-09-11

**Authors:** A Bogachev-Prokophiev, S Zheleznev, A Pivkin, S Lavinyukov, I Ivanov, V Nazarov, A Karaskov

**Affiliations:** 1 State Research Institute Blood Circulation Pathology, Novosibirsk, Russian Federation

## Background

Use of irrigation catheters increases the power supply energy to create transmural injury [Jais P., Shah D.C., Haissaguerre M. et al., 2000]. However, these data are based on the catheter procedures and concomitant monopolar endocardial ablation [Deneke T., Khargi K. et al., 2007]. The benefits of using irrigation during bipolar concomitant ablation has no reflection in the literature.

## Methods

from 2007 to 2012, 407 patients (mean age 58.2 ± 10.4 years) underwent valve or coronary procedure and concomitant bipolar RF ablation for atrial fibrillation (AF). In 152 (37,3%) patients was used irrigated bipolar clamp Medtronic CardioBlate (IB group) and dry bipolar clamp AtriCure Isolator Synergy (DB group) in 255 (62,7%) cases. Most of the patients in both groups had a long standing persistent AF. There were no significant differences between groups. The follow-up data was analyzed for 36,3 ± 14,6 months. Rhythm disturbances evaluated by 24 hours Holter.

## Results

No significant differences in ablation time and aortic cross-clamping: 25.2 min in BI group and 27.6 min in BD group (p = 0.271); 82.7 min in BI group and 78.3 min in BD group (p = 0.163). There was no difference in hospital mortality between the groups. Specific ablation complication (pulmonary vein perforation) occurred in 1 patient in each group: 0.66% (BI group) and 0.39% (BD group) (p = 0.576). At latest follow up freedom from AF was 81.3% in the BI group and 84.7% in the BD group (p = 0.121). Atrial flutter occurred in 5.9% (BI group) and in 1.9% (BD group) (p = 0.047). All patients with atrial flutter were underwent catheter ablation.

## Conclusions

Comparative analysis revealed no any benefits of using irrigation during concomitant bipolar ablation.

